# Thymus vulgaris essential oil: chemical composition and antimicrobial activity

**Published:** 2014

**Authors:** O Borugă, C Jianu, C Mişcă, I Goleţ, AT Gruia, FG Horhat

**Affiliations:** * Department of Ophthalmology, Victor Babeș University of Medicine and Pharmacy, Timișoara, Romania; ** Department of Food Science, Faculty of Food Processing Technology, Banat’s University of Agricultural Sciences and Veterinary Medicine, Timișoara, Romania; *** Department of Management, Faculty of Economics and Business Administration, West University of Timișoara, Timișoara, Romania; **** Center for Transplant Immunology, Timișoara County Hospital, Timișoara, Romania; ***** Department of Microbiology, Victor Babes University of Medicine and Pharmacy, Timisoara, Romania

**Keywords:** thyme, essential oil, GC-MS analysis, antimicrobial activity

## Abstract

The study was designed to determine the chemical composition and antimicrobial properties of the essential oil of Thymus vulgaris cultivated in Romania. The essential oil was isolated in a yield of 1.25% by steam distillation from the aerial part of the plant and subsequently analyzed by GC-MS. The major components were p-cymene (8.41%), γ-terpinene (30.90%) and thymol (47.59%). Its antimicrobial activity was evaluated on 7 common food-related bacteria and fungus by using the disk diffusion method. The results demonstrate that the Thymus vulgaris essential oil tested possesses strong antimicrobial properties, and may in the future represent a new source of natural antiseptics with applications in the pharmaceutical and food industry.

## Introduction

The genus Thymus, member of the Lamiaceae family, contains about 400 species of perennial aromatic, evergreen or semi-evergreen herbaceous plants with many subspecies, varieties, subvarieties and forms [**1**]. In Romania, the Thymus genus contains one species cultivated as aromatic plant (Thymus vulgaris) and other 18 wild species [**2**]. T. vulgaris (thyme), locally known as “cimbru”, is widely used in the Romanian folk medicine for its expectorant, antitussive, antibroncholitic, antispasmodic, anthelmintic, carminative and diuretic properties.

Various studies have aimed to investigate the chemical composition and biological properties of the T. vulgaris essential oil (EO). According to European Pharmacopoeia 5.0 (Ph. Eur. 5.0) [**3**], the minimum content of EO in T. vulgaris is 12 mL/kg, but the chemical composition shows variations, six chemotypes being mainly reported, namely geraniol, linalool, gamma-terpineol, carvacrol, thymol and trans-thujan-4-ol/terpinen-4-ol [**4**,**5**]. Both the isolation yield and the chemical composition of the EOs are dependent on a number of factors, such as the environment, growth region and cultivation practices [**6**]. In addition to the flavoring properties determined by the constitutive active ingredients, the thyme EO exhibits significant antimicrobial activity [**4**,**7**-**9**] as well as strong antioxidant properties [**2**,**8**].

The aim of this study is to determine the chemical composition together with the antimicrobial properties of the EO of T. vulgaris cultivated in Romania, in order to identify new sources of natural antiseptics with applications in the pharmaceutical and food industry.

## Materials and methods

**Raw material**. Thyme was harvested during the flowering season (July 2012) from the area around the Broşteni commune – Mehedinţi County, Romania. The plant material was dried in well-ventilated areas, sheltered from direct sunlight and then stored in double-layered paper bags at temperatures of 3-5°C until processing. A voucher specimen (V.FPT-451) was deposited in the Herbarium of the Faculty of Pharmacy, “Victor Babeș” University of Medicine and Pharmacy, Timișoara, Romania.

**Isolation of essential oils**. The EO was obtained by hydrodistillation, according to Ph. Eur. 5.0 [**3**], by using a modified Clevenger apparatus (with the EO collection area cooled to prevent the emergence of artifacts). The EO was dried on anhydrous sodium sulfate (Sigma-Aldrich Chemie GmbH) and stored in a tightly sealed brown glass bottle at 0-4°C for testing.

**Gas chromatography-mass spectrometry**. Samples were analyzed by gas chromatography using a HP6890 instrument coupled with a HP 5973 mass spectrometer. The gas chromatograph is equipped with a split-splitless injector and a Factor FourTM VF-35ms 5% fenil-methylpolysiloxane, 30 m, 0.25 mm, 0.25 μm film thickness capillary column. Gas chromatography conditions include a temperature range of 50 to 250°C at 40°C/min, with a solvent delay of 5 min. The injector was maintained at a temperature of 250°C. The inert gas was helium at a flow of 1.0 mL/min, and the injected volume in the splitless mode was 1 μL. The MS conditions were the following: ionization energy, 70 eV; quadrupole temperature, 100°C; scanning velocity, 1.6 scan/s; weight range, 40-500 amu.

The percent composition of the volatile compounds was calculated. The qualitative analysis was based on the percent area of each peak of the sample compounds. The mass spectrum of each compound was compared with the mass spectrum from the NIST 98 spectrum library (USA National Institute of Science and Technology software).

**Determination of antimicrobial activity**. Thyme EO was tested on 7 common food-related bacteria and fungus: Staphylococcus aureus (ATCC 25923), Pseudomonas aeruginosa (ATCC 27853), Salmonella typhimurium (ATCC 14028), Escherichia coli (ATCC 25922), Klebsiella pneumoniae (ATCC 13882), Enterococcus faecalis (ATCC 29212) and Candida albicans (ATCC 10231), using the disk diffusion method as previously described [**10**]. Briefly, a suspension of the tested microorganism (10^6 cells/mL-1) was spread on the solid media plates (Mueller-Hinton agar for bacteria and Sabouraud cloramphenicol agar for fungi). The paper discs (Whatman No 1 filter paper - 6 mm diameter) were impregnated with 5, 10, 15 and 20µL EO and placed on the inoculated agar. The plates inoculated with bacterial strains were incubated for 24 h at 37°C and 48 h at 30°C for fungi, respectively. As positive controls, ciprofloxacin (30 µg/disk) and cephalexin (10 µg/disk) were used for bacterial strains and fluconazole (10 µg/disk) for fungi. After incubation, the diameter of the zone of inhibition was measured in millimeters. Each test was performed in triplicate on at least three separate experiments.

**Statistical analysis**. The statistical analysis was performed by using SPSS Version 21 (IBM Corp., NY). The mean inhibition zone for each group of nine observations was compared with the value of the disc diameter (6 mm) using the t-test. The GLM procedure was used to conduct a two-way analysis of variance (ANOVA) on the inhibition zones. The type of microorganism and amount of essential oil were used as factors in the full factorial model. Post-hoc tests for each amount of essential oil were conducted by using Tukey’s HSD method, in order to compare the effect on different types of microorganisms.

## Results and Discussion

The isolation yield was 1.25% (v/w), based on dry plant material and confirmed that the plant analyzed meets the requirements of pharmaceutical quality for thyme as EO source [**3**]. The chemical composition determined by GC/ MS is presented in **Table 1**. Fifteen components representing 99.91% of the total detected constituents were identified. The major components were p-cymene (8.41%), γ-terpinene (30.90%) and thymol (47.59%), which suggests that the EO analyzed belongs to the thymol chemotype in agreement with those previously reported in Romania [**2**]. The other components were present in a total amount of less than 13.01%. The chemical composition of the EO analyzed by us is very different from that previously reported in Morocco and Spain for the same species of thyme [**11**,**12**]. Similar studies in Poland, Iran, Spain and Italy, respectively, reported as major compounds in the T. vulgaris EO p-cymene, γ-terpinene and thymol [**4**,**13**-**15**]. These differences can be attributed to a large extent to the different chemotypes mentioned above [**4**,**5**,**13**].

**Fig. 1:**
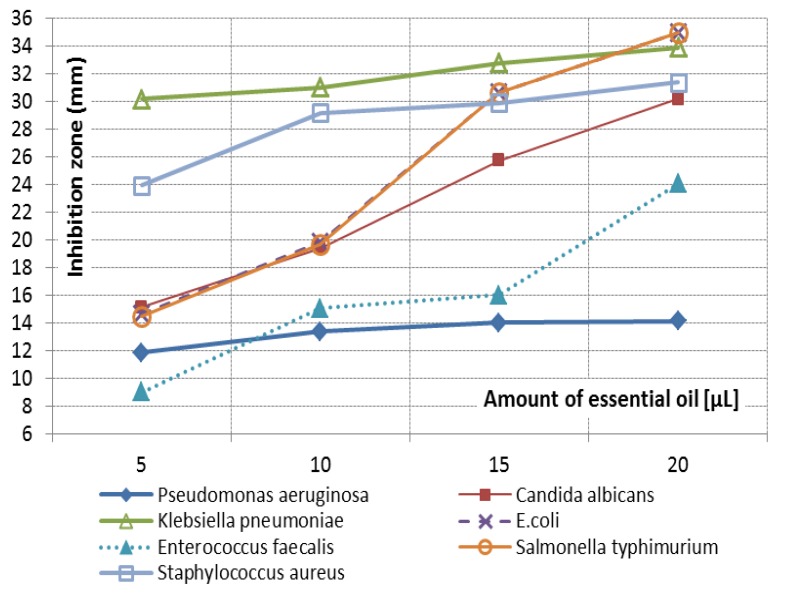
The antimicrobial activity of thyme oil, at different amounts, expressed as a mean inhibition zone for each of the nine repeated measurements

The antimicrobial activity of thyme oil against 7 common food-related bacteria and fungus tested is presented in **Table 2**. The null hypothesis that the inhibition zone is equal to the disc diameter (6 mm) was rejected for each microorganism at every amount of essential oil with a high significance level (p = 0.00). The main finding of the ANOVA analysis is a strong interaction effect between the type of microorganism and the amount of essential oil (p = 0.00). The highly significant interaction effect adds difficulty in drawing general conclusions on the main effects, even if the two factors are also highly significant (p = 0.00). For example, K. pneumoniae has the highest inhibition zone overall but for the amount of 20 [μL], where E. coli and S. typhimurium have higher values. In order to compare more thoroughly the effect of T. vulgaris on each microorganism (**Fig. 1**), the results of multiple comparisons, at each oil amount, has to be considered. Tukey’s HSD test reveals that the only microorganisms with non-significant differences in the antimicrobial effect are S. typhimurium and E. coli at all oil amounts, and S. typhimurium, E. coli and C. albicans at 10 [μL]. The observed p-value for the pairwise differences in the above-mentioned cases does not pass acceptable significance levels, being larger than 0.4. All the other pairwise differences are highly significant (p = 0.00).

**Table 1:** Chemical composition of thyme EO

No.	RT (min)	Area % of total	Constituents*
1	5.39	1.06	alpha-Thujene
2	5.63	1.07	alpha-Pinene
3	6.89	0.37	beta-Pinene
4	6.97	1.53	beta-Myrcene
5	7.53	0.33	alpha-Phellandrene
6	7.77	3.76	Carene<δ-2->
7	8.04	0.29	D-Limonene
8	8.26	0.21	beta-Phellandrene
9	8.46	8.41	para-Cymene
10	8.96	30.90	gamma-Terpinene
11	9.48	0.47	Terpineol
12	12.55	0.46	Terpinen-4-ol
13	16.17	47.59	Thymol
14	17.32	2.68	Caryophyllene
15	19.03	0.78	Cyclohexene, 1-methyl-4-(5-methyl-1-methylene-4-hexenyl)
Total		99.91%	
*Constituents presented in the order of elution from the VF 35 MS column.

The inhibition of the growth of E. coli, K. pneumoniae, S. aureus, P. aeruginosa and E. faecalis was previously reported [**4**,**7**,**9**] along with the efficacy against C. albicans [**9**,**16**,**17**] and S. typhimurium [**4**,**9**], respectively. In contrast, some studies report the inefficiency of thyme EO against E. coli [**16**,**17**], S. aureus [**16**] and K. pneumoniae [**16**].

**Table 2:** Effects of thyme oil against bacteria expressed by the mean sizes of the inhibitory zones

Test microorganism	Amount of essential oil [μL]			
	5	10	15	20
*Staphylococcus aureus* ATCC 25923	23.93 ± 0.33	29.2 ± 0.6	29.9 ± 0.35	31.4 ± 0.47
*Salmonella typhimurium* ATCC 14028	14.49 ± 0.34	19.71 ± 0.39	30.68 ± 0.33	34.94 ± 0.22
*Pseudomonas aeruginosa* ATCC27853	11.82 ± 0.27	13.34 ± 0.33	14 ± 0.22	14.13 ± 0.19
*E. coli* ATCC 25922	14.63 ± 0.36	19.82 ± 0.41	30.67 ± 0.31	34.99 ± 0.19
*Klebsiella pneumoniae* ATCC 13882	30.21 ± 0.12	31.02 ± 0.31	32.79 ± 0.24	33.93 ± 0.14
*Enterococcus faecalis* ATCC 29212	8.99 ± 0.15	15.06 ± 0.15	15.99 ± 0.18	24.06 ± 0.15
*Candida albicans* ATCC 10231	15.14 ± 0.38	19.43 ± 0.55	25.74 ± 0.24	30.2 ± 0.17

The inhibitions are expressed in mm and include the diameter of the paper disc (6 mm). Data distributions were expressed as mean values and standard deviations (SD) (n = 9). Ciprofloxacine and cephalexine (for bacterial strains) and fluconazole (for fungi), respectively, were used as positive controls.

The antimicrobial activity of EOs depends on their chemical constituents. Apparently, the antimicrobial activity of the EO analyzed is related to the presence of phenolic compounds (thymol) and terpene hydrocarbons (γ-terpinene), respectively [**4**,**7**,**18**]. p-Cymene, the third major element according to percentage, does not show antibacterial efficacy when used alone [**7**], synergistic effects being however attributed to it in relation to thymol and γ-terpinene, respectively [**19**,**20**], which might represent another cause of the antimicrobial activity recorded. On the other hand, a number of studies have shown that EOS exhibit stronger antimicrobial activity than that of their major constituents or their mixtures, respectively [**21**,**22**], which suggests synergistic effects of the minor components, but also the importance of all components in relation to the biological activity of EOs.

## Conclusions

The results demonstrate the effectiveness of thyme EO against the food-related bacteria and fungus tested. The synergism, antagonism and additive effects, respectively, of the EOs components require further research to elucidate the mechanisms underlying their biological activity, for the purpose of accessing new natural antiseptics applicable in the pharmaceutical and food industry.
